# Safety: a proportionate approach in an uncertain application

**DOI:** 10.1098/rsta.2023.0405

**Published:** 2024-08-26

**Authors:** Omar Afify

**Affiliations:** ^1^ Office of the Chief Engineer, UKAEA, Culham Campus, Abingdon OX14 3DB, UK

**Keywords:** step, safety case, hazards, regulation, fuel cycle

## Abstract

Fusion is inherently safer than fission due to the absence of nuclear chain reactions. However, operating fusion power plants will not be risk free. There will still be numerous hazards that will need careful management in order to safely build, operate and ultimately decommission a fusion power plant. Ensuring a robust safety demonstration that covers all radiological and non-radiological hazards is therefore vitally important for the future permissioning and consenting of fusion power plants. The safety case for the STEP prototype plant will be developed in line with a set of safety philosophies, safety functional requirements and design safety principles to ensure that the safety case production process is consistent and robust.

This article is part of the theme issue ‘Delivering Fusion Energy – The Spherical Tokamak for Energy Production (STEP)’.

## 1. Introduction to fusion hazards

Fusion is the process where two light atomic nuclei, e.g. deuterium and tritium (both variants of hydrogen), fuse together to form a single heavier nucleus, releasing massive amounts of energy. This is the opposite of nuclear fission, where a heavy atomic nucleus (typically uranium-235) is split into smaller nuclei. Two or three neutrons are also released in the process, and these collide with other uranium nuclei to cause further fission reactions—a chain reaction.

The fusion process is therefore inherently safer than fission due to the absence of these chain reactions. However, operating fusion power plants will not be risk free, as for any other industrial plants. There will still be numerous hazards that will need careful management in order to safely build, operate and ultimately decommission a fusion power plant. Safety assessments are therefore crucial in understanding the hazard potential that can arise from the inventories present within a fusion facility. These can be grouped under the following categories [1]:

— Energy inventory
  - In-vessel fuel energy—energy that could be released from the residual fuel in the vacuum vessel.
  - Magnetic energy—failure of the magnets could result in the discharge of its energy into the wall of the vacuum vessel or other structural materials.
  - Residual heat in the plasma facing components.
— Radioactive material inventory
  - Tritium.
  - Radioactive dust and corrosion products—due to the corrosion or erosion of the vacuum vessel wall or other structural materials.

The safety assessments will therefore need to consider these inventories within the plant, as well as how the plant will handle routine releases and how it behaves under postulated accident scenarios. The key accident scenarios during operations can be broadly grouped as follows:

— Loss of coolant or breeder (in- and ex-vessel)
— Loss of coolant flow
— Loss of vacuum (either in the cryostat or vacuum vessel)
— Magnet events (e.g. quench)
— Plasma events (e.g. disruptions)
— Fuel cycle process faults
— Faults during maintenance and/or intervention activities
— Exposure to activated components, liquids or gases
— Exposure to chemicals, toxic and/or hazardous substances
— Internal and external events (naturally occurring and man-made hazards)
— Industrial hazards (e.g. high voltages, flammable substances, cryogens and chemicals).

## 2. Development of a fit for purpose safety case approach

Ensuring a robust safety demonstration that covers all radiological and non-radiological hazards is vitally important for the future permissioning and consenting of fusion power plants. The safety case for the STEP prototype plant (SPP) will be developed in line with a set of safety philosophies, safety functional requirements and design safety principles (DSPs) to ensure that the safety case production process is consistent and robust.

### (a) STEP safety philosophies

#### (i) Safety and project risk are distinctly separate

The safety of the project will not be compromised; however, the approach can be flexible, provided the safety goals are achieved at an appropriate point.

#### (ii) Claims will be made on a few robust measures rather than multiple complex systems

To address the uncertainty inherent in this new technology and the effort required to substantiate the associated engineering, claims will be made on a few robust measures rather than multiple complex systems, e.g. by claiming robust containment systems rather than multiple lower reliability claims on complex in-vessel components. This approach is necessary for a first of a kind fusion power plant, which is a novel and complex machine, with environments that make substantiation of materials difficult. It will also result in a simplified safety case.

#### (iii) Clear distinction will be made between claims relating to asset protection and those required to ensure safety

There will be a significantly higher acceptance of risks for asset protection faults, in comparison with risks to human safety or environmental protection. As the aim is to simplify the safety case, it is anticipated that asset protection measures (e.g. control and instrumentation systems for machine protection) will not be claimed in the safety case.

#### (iv) The need for off-site emergency evacuation will be minimized

The SPP will minimize the off-site impact of any emergency arrangements required. In particular, the intent is to eliminate the need for off-site emergency evacuation.

### (b) Top-level safety functional requirements

The top-level safety functions are specific objectives that must be accomplished in the interests of safety during normal operations and/or during fault or accident conditions. The safety functions for the SPP have been developed using the International Atomic Energy Agency (IAEA) safety functions as a basis, while also recognizing that non-radiological hazards also need to be managed. The top-level safety functions for the SPP are as follows:

— Ensure adequate protection for workers, the public and the environment against the release of radioactive materials due to accident scenarios and during normal operations.
— Ensure that the exposure of personnel to direct radiation is as low as reasonably practicable (ALARP).
— Ensure that significant non-radiological hazards are minimized as far as reasonably practicable.

The top-level safety functions are specified with minimal reference to the physical means by which they will be delivered, e.g. containment of radioactive material. As the safety functional requirements are developed throughout the design, progressively more detailed and specific safety functions will be identified to achieve the top-level safety functions. This development will continue until specific safety functional requirements have been produced. Safety functions may be delivered by engineered measures or supported by human actions.

### (c) Design safety principles

DSPs are used in helping to judge whether reducing risks to ALARP is achieved. Priority is given to achieving an overall balance of safety rather than strictly satisfying each principle; the principles themselves should be met so far as is reasonably practicable and considered within the appropriate context (e.g. by considering those that are relevant to a particular design decision being evaluated). The design must first be viable and practicable when judging whether risks are ALARP. A set of DSPs have been defined to guide a safe-by-design approach to the SPP, minimizing the risk to operators and the public. A selection of the DSPs is presented below.

— *Public risk*: Risks associated with fault scenarios will be minimized such that the potential for public evacuation is minimized. A stretch target will be set of not requiring an off-site plan.
— *Limitation of risk to individuals*: Measures for controlling risks must ensure that no individual bears an unacceptable risk of harm.
— *Inventory*: The design will aim to minimize the inventory of radioactive or other hazardous material where reasonably practicable; in particular, the inventory that may potentially be released during fault scenarios should be minimized.
— *Stored energy*: Stored energy within systems will be minimized as far as reasonably practicable to limit the potential consequences of an uncontrolled release (e.g. magnetic, electrical and chemical).
— *Environmental release*: Containment and associated systems will be designed to minimize radioactive and hazard material releases to the environment in normal operation and fault scenarios.

### (d) Fusion safety case pillars

To ensure the safety case can be made for the SPP, the fusion safety case pillars that are essential for early safety case involvement have been identified and discussed below.

#### (i) Normal operation hazards and facility zoning philosophy

To ensure normal operational exposure to ionizing radiation is minimized, exposure pathways for public, workers and other site personnel for routine releases need to be analysed, as well as the doses from direct radiation hazards. Consequences from non-radiological hazards will also be considered, e.g. electromagnetic radiation, magnetic fields, lithium and other chemicals. Hazards during maintenance will also be assessed.

#### (ii) Control of stored energy

Hazard management strategies will be developed for stored energy sources such as plasma, magnets and cryogens. These energy sources need to be managed to ensure that failures cannot result in injury to personnel directly, or as a result of a subsequent release of radioactive or hazardous material, e.g. due to damage to a confinement boundary. The different types of events and their consequences will be assessed to ensure that adequate protection measures are in place.

#### (iii) External hazards strategy

External hazards that could affect the safety of the SPP will be identified, characterized and analysed based on the risk the external hazard poses, i.e. the consequences and frequency will be assessed to determine the required withstand of the facility barriers/systems. To avoid unnecessary qualification of buildings, plant and equipment, other hazard management strategies will be considered, such as minimizing hazardous inventories, providing segregation and separation and the use of passive storage.

#### (iv) Containment strategy

Suitable and sufficient containment barriers are required for radioactive and other hazardous materials to ensure risks to personnel and the public during normal operations and fault scenarios are ALARP. All forms of radioactive/hazardous inventory will be considered, e.g. tritium, activated dust, activated coolants, as well as various plant configurations, e.g. commissioning, operations and maintenance. The containment function is generally provided by a succession of strong physical barriers, together with active systems such as isolation valves and ventilation systems. The need for over-pressure protection measures into closed systems will also form part of this consideration, subject to the output of further safety assessments which will define their requirements.

#### (v) Development of safety claims

The safety claims will evolve as the programme matures, and information becomes available from the successive stages of the programme. Operational phases are devised to give a staged approach to increasing hazard potential as confidence develops. One example of this approach is that tokamak component materials will be validated in service because the relevant modelling and assumptions cannot be fully validated prior to use. Another example will be to prove remote maintenance techniques in a low-hazard environment during early phases rather than in a high-activation environment where tritium is present. Therefore, the projects confidence will grow through increased understanding.

#### (vi) Safety input to tokamak and site layout decisions

The tokamak design and layout will be optimized to minimize the potential for hazards to occur and ensure that internal and external hazards such as fire, explosion and flooding are adequately managed. The SPP site layout will be optimized to prevent interactions between facilities and minimize the risks to members of the public. The location of hazardous facilities and interactions between different facilities and transport routes will be assessed, along with risks posed by external hazards.

#### (vii) Waste management and decommissioning plan

The SPP design will consider provisions to facilitate waste management and the future decommissioning of the facility from the concept design stage. STEP aims to follow the waste hierarchy for both radioactive and non-radioactive wastes, i.e. targeting waste prevention and reduction and prioritizing recycling over disposal. Activation and contamination of structural materials will be minimized to allow recycling routes to be developed.

## 3. Development of key fault sequences and hazard management strategies

The complex nature of a fusion power plant means that trade-offs have had to be made to ensure that a viable design is achieved. However, throughout the development of the design, safety has been and will remain a key aspect of the decision-making process. Where certain decisions or trade-offs have implications for safety, specific focus has been placed on assessing the relevant issues to guide the design and ensure adequate safety measures are incorporated in the future. For example, decisions around the placement and coverage of the limiters considered the safety impacts of a loss of coolant accident (LOCA) from ultra-high heat flux transient events. This limited the choice of coolants, with gases (e.g. helium) preferable to liquids (e.g. water) due to less stringent mitigation system requirements in the event of an LOCA [2].

To embed safety into the design from an early stage, safety assessments have focused on design-driving issues. At this stage, the focus has been on key tokamak faults such as LOCAs [2]. With this in mind, a preliminary hazard log (or schedule) has been developed, taking learning from existing UKAEA fusion research facilities and safety documentation for international fusion projects (such as ITER and EU DEMO), while capturing STEP-specific hazards identified through various hazard identification exercises and reviews undertaken to date. These are then collated into a series of high-level groups based on common safeguards or consequences. These faults are then brought forward into the fault schedule for further assessment in the safety case.

The purpose of the fault schedule is to provide a summary of each fault group with the key findings of the corresponding safety assessment, such as postulated IEF and consequences should the fault occur. As the STEP design is still at a relatively early stage, specific details concerning safety measures are not yet available. The schedule therefore presents high-level hazard management strategies and potential (or candidate) safety measures that could protect against the fault. Future design development and optioneering will then confirm the claims that will be made and define the specific performance requirements placed on the different safety measures.

Faults are not broken down into further detail based on the type of initiating event, as the general strategy is to claim robust containment boundaries to protect personnel rather than systems that prevent initiators resulting in loss of coolant, plasma control, etc.

A key part of the fault schedule development is the categorization of the high-level safety functions according to the estimated initiating event frequency (IEF) and consequences. Safety functions are specific objectives that must be accomplished in the interests of safety during normal operations and/or during fault or accident conditions, an example of which can be ‘confinement of radioactive material’. These high-level safety functions can be broken down into more specific safety functions, and as the design of the facility develops, the progressive breakdown of safety functions should continue until they are clearly attributable to the safety measures that will be assigned to deliver these safety functions. Depending on the complexity of the fault sequence, more than one safety function may need to be delivered to manage the fault.

The safety function category is then used to provide guidance on the required number of safety measures and their classification. The categorization of safety functions is based on both the consequence of failing to deliver the safety function and on the frequency of demand placed on the safety function. The consequence of failing to deliver the safety function represents the unmitigated effective radiation dose that could be received by a person either on-site or off-site.

The safety function categorization scheme is based on three categories:

— *Category A*: Any function that plays a principal role in ensuring radiological safety.
— *Category B*: Any function that makes a significant contribution to radiological safety.
— *Category C*: Any other safety function contributing to radiological safety.

**Figure 1.**
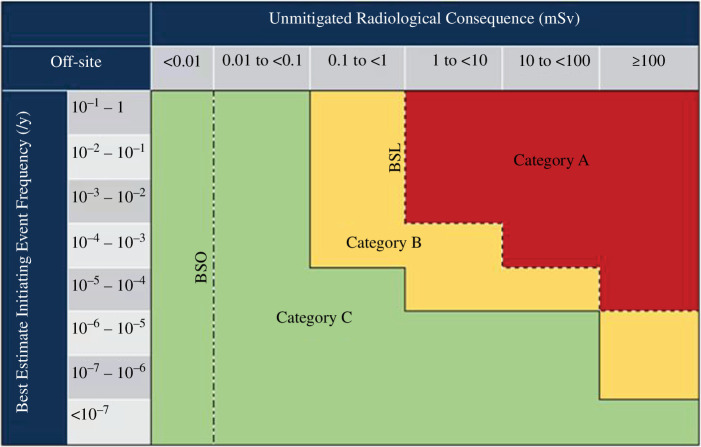
Initial radiological safety function categories (public dose).

In terms of categorization, the UKAEA design basis analysis guidance shows that for public doses greater than 1 mSv and an IEF higher than 10^−3^/yr, the resulting category is category A (figure 1). Faults that lie within this highest category of safety function (category A) will require robust and reliable safety measures to ensure that the overall risk from that fault is ALARP.

**Figure 2.**
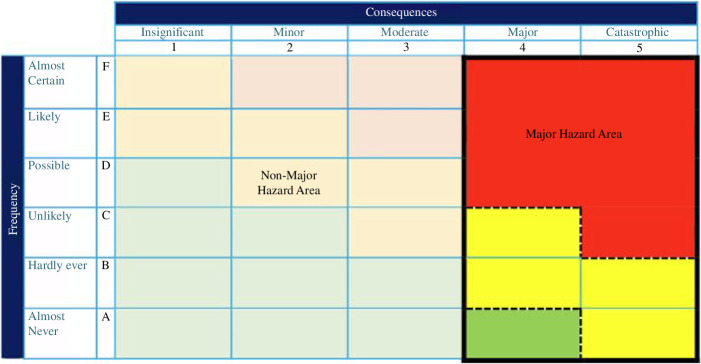
Major hazard area (adapted from the non-radiological hazard assessment guidance).

Significant non-radiological consequences are also expected from some of the fault groups depending on their escalation potential and their mitigation, e.g. faults involving lithium reactions leading to hydrogen generation and flammable/explosive atmospheres. Categorizing these will be carried out in accordance with the UKAEA Non-Radiological Major Hazard Assessment guidance and follows a similar process to that described earlier, where faults with the potential for major or catastrophic consequences (figure 2) requiring robust and reliable safety measures to ensure that the risks are ALARP.

Hazards and candidate measures have also been captured for other aspects such as normal operations, maintenance and the fuel cycle, as well as internal and external hazards which influence building and site layout. This ensures that safety case aspects are being considered within the design at multiple levels. This systematic approach will ensure that the safety case development is integrated with the design process to ensure that suitable and sufficient safety measures are provided to reduce risks to ALARP.

## 4. Fuel cycle safety and environment aspects

The STEP fuel cycle [3] contains many safety, environmental and security challenges:

— The fuel cycle is a series of pressurized vessels and cascades containing radiological and flammable species.
— The majority of the tritium inventory will be stored on depleted uranium (DU) beds. The use of DU beds introduces an industry-recognized hazard but also containment, public image, security and regulatory considerations.
— Seeded impurity by-products could introduce small quantities of chemically active and hazardous species.
— Inherited safety considerations from interfaces, specifically the selection of liquid lithium and heavy water coolants.
— Expect multifarious end-of-life low-grade waste to be generated including tritiated working fluids, process equipment and plant.
— High volume of tritiated water on-site is a significant radiological hazard requiring management but lends to a fuel cycle architecture with mature technologies supporting low nominal gas discharge.

Some such as the handling of tritium are intrinsic while others such as the presence of large volumes of tritiated heavy water on-site are inherited from key wider plant interfaces. The fuel cycle will contain flammable, explosive and hazardous species, including tritium, activated impurities and lithium. Consideration of these aspects early in the design concept phase is key to developing a safe and successful fuel cycle design.

The management and control of tritium within the STEP plant is a critical function necessary to provide safety, environmental and security assurances, as well as process control. Tritium accountancy, whereby tritium inventories within the fuel cycle are tracked and reported for proliferation and security purposes, is particularly challenging due to the continuous nature of the fuel cycle, the long operational periods, the high rate of tritium consumption and production and the retention of tritium in process equipment. In STEP, the plant tritium inventory will be tracked by monitoring tritium imports, exports, consumption, production and wastes. Material balance areas will be defined, but highly detailed tracking of tritium within the fuel cycle may not be possible without accumulating large measurement uncertainties. Accountancy must therefore form only one layer of a defence-in-depth approach to protecting tritium, which applies proportionate measures to assessed security risks. Real-time management of tritium must also be developed and used which will rely upon a suite of measurement techniques. The STEP programme intends to draw upon technical capability and experience within the supply chain in providing analytical instrumentation by collaboration with suppliers to adapt, where possible, existing mature measurement technologies for tritium applications.

As part of instilling a design culture and approach of design-for-safety, a series of safety sprints were held with safety consultants from an engineering delivery partner from the supply chain. The fuel cycle modelling capability development and safety review sprint programme helped ensure that the relative and intrinsic safety of different technology options were considered ahead of the technology down-selection process. It also helped develop an appreciation of how one sub-system could impact adjacent and downstream sub-systems. These safety sprints included a series of block-level hazard identification workshops before using relative risk assessments of technology candidates to identify the comparatively safer options. The safety sprint helped to identify preventative measures that could be introduced to mitigate safety risks and ensure safety influences key fuel cycle technology and architectural decisions. Identified preventative measures output by the safety sprints has provided the sub-system owners with a catalogue of design actions for consideration which will help ensure safety can be designed in as the design takes form at a sub-system level. Furthermore, understanding of safety risks in adjacent systems will help with fuel cycle architecting to mitigate against common mode system failures.

Further to using the safety sprints to engender a culture and design philosophy of design-for-safety, there has been close integration with the environment team. The environment team, supported by a team of subject matter experts from an engineering delivery partner from the supply chain, led training of the STEP fuel cycle team on a best available techniques (BAT) approach. The BAT and safety sprint assessments largely converged with existing thinking, e.g. for the requirement to minimize system tritium inventory and the need for overarching systems for the purposes of protecting workers and the environment from the intrinsic risks associated with tritium handling. Sub-systems that will help with tritium handling include the following:

— Water detritiation system, atmospheric detritiation system, trace tritium recovery and gas detritiation system all recover tritium from process water, process gas and room air streams prior to discharge to stack. Technologies with high detritiation factors were selected to ensure that the maximal amount of tritium is recovered and residual radioactivity in discharges is minimized.
— Coolant detritiation systems shall be incorporated into the STEP fuel cycle to recover tritium from the heavy water and helium coolant loops such that tritium levels within the coolants are maintained below agreed limits and safety thresholds.
— Tritium management and control systems shall be integrated across the fuel cycle design. This will include control to re-route gaseous discharges, if the activity exceeds discharge limits under certain scenarios, and monitoring to ensure inventories remain within predefined limits and adhere to the plant safety case.
— A containment sub-system shall be incorporated into the STEP fuel cycle to capture, contain and return permeated and/or leaked tritium flows. The containment system will use a mixture of pressure differentials and purge gases to minimize losses from the secondary system. As the containment system develops during the design definition phase, one key aspect to consider is careful crafting of the design standards and codes to minimize, where feasibly possible, common mode failures that lead to primary and containment system rupture.
— A fire management and protection sub-system shall be incorporated into the STEP fuel cycle to address and contain the spread of flame and fumes in the event of a tritiated fire. The fire strategy needs to include how to manage not only tritium gas fires but also fires involving the lithium breeder.
— An isolated drainage and water containment system will be incorporated into the STEP fuel cycle facilities to contain any water leaks and to prevent mixing with the wider plant storm drains without prior specialist and prolonged processing.

Initial studies using preliminary discharge modelling have been undertaken using trial values of potential steady-state nominal gaseous fuel cycle discharge levels. These values correspond to potential sizing cases for the outer fuel cycle blocks. Although failure cases have yet to be modelled and can be expected to ultimately size the fuel cycle, first results show a large margin relative to compliant dosage levels in the local environment.

Further to the tritium handling risks, there is a need to manage by-products of seeded impurities including several different radioisotopes of argon and xenon. In addition, fractions of chemically active species shall also be generated, which may readily form acids and hydrides. Seeded impurity recycling with fractional purging will minimize radiological discharge while mitigating the accumulation of large quantities of chemically active, tritiated and radiological species caused by extensive repeat neutronic bombardment of already activated by-products. The primary focus will be the removal of long-lived isotopes of argon.

The seeded impurity management strategy is still under development, and it is recognized that there remains a significant challenge within the fuel cycle regarding optimization of the sequencing for separating and removing the large range of different exhaust species. Careful consideration is required to ensure that the technology deployed for filtering out one species is not poisoned or otherwise adversely impacted by the presence of other exhaust species. Careful consideration is also required to ensure that handling procedures are in place to protect workers from parts of the fuel cycle that contain activated seeded impurity by-products and traces of tritiated acids. Protection to workers includes ensuring containment but also using robust design principles to maximize product maturity via design. This should help deliver high-life design solutions that minimize the number of maintenance activities that require breaking into the system, increasing the risk of exposure and wider facility contamination.

## 5. Areas for future research and development

Research and development in fusion is extensive and is at the heart of what UKAEA does. The emphasis behind this lies in commercializing the technology on a large scale and includes areas such as material science, magnet technology and fuel self-sufficiency among many others. From a safety perspective, this includes safety assessment tool development to allow a better understanding of how the plant behaves during normal and abnormal conditions.

Evaluating risk requires prior knowledge and understanding of the probabilities of the initiating events and the subsequent performance of the safety systems. Currently, there is a gap in component failure rate data for evaluating accident probabilities as many fusion-specific systems have no data (as they are new) and hence cannot be accurately assigned failure rates [1]. For STEP and other future fusion power plants, the consequences of accidents where safety systems fail to act or are impaired need to be established. Taking an ingress of cooling water into the vacuum vessel [2] as an example, there will need to be an analysis of the resultant pressure within the vacuum vessel should safety measures (such as bursting discs) fail and if this pressure has the potential to damage the vessel. To that end, various assessments have been conducted to investigate the possible consequences of a coolant ingress into the vacuum vessel. Initial calculations have been supplemented by *in silico* modelling using the MELCOR code, which is an integrated engineering-level thermal hydraulics computer code that models the progression of accidents in fusion power plants.

Further work is also underway to model other important safety aspects, such as atmospheric dispersion behaviours of tritium, especially accounting for buoyancy factors if the release is during a fire scenario, coupled with wind tunnel experimental data to reduce the uncertainty of atmospheric dispersion and to validate existing computer simulations. These will drive the design of the SPP, as they will impact decisions around confinement boundaries and selection of safety systems, which are needed for a robust safety analysis and the subsequent engineering substantiation.

## 6. Regulation of fusion facilities

The Health and Safety at Work Act 1974 places a legal requirement on employers to ensure that all reasonably foreseeable risks to their employees and to any persons off-site who may be impacted by their undertakings are reduced to a level that is ALARP. Experimental fusion facilities are currently regulated by the Health and Safety Executive (HSE) against the general duties in the Health and Safety at Work Act 1974, the Management of Health and Safety at Work Regulations 1999 and the more specific requirements for workers and others protection in (primarily) the Ionising Radiation Regulations 2017. Legislation that determines environmental protection regulation is enforced by the Environmental Agency (EA) in England. Regulators in each of the other nations in the UK have the responsibility for carrying out this same function.

The UK Government has confirmed the decision for the HSE and EA to retain this responsibility for future fusion energy facilities in the UK through the Energy Act 2023, which also amends Section 1 of the Nuclear Installations Act 1965 so that it does not apply to fusion energy facilities and confirming that they will not require a nuclear site licence. Internationally, the USA has also taken a similar approach to that of the UK Government, with the US Nuclear Regulatory Commission voting unanimously to regulate near-term fusion energy systems under Part 30 of the Code of Federal Regulations, rather than using Part 50 of that code which regulates the fission industry. These decisions recognize the lower hazard potential inherent to fusion when compared to fission and allows the industry to develop technologies according to clear requirements and guidelines. The UKAEA, along with national and international partners, has also been supporting the IAEA in developing technical documents featuring experiences for consideration in fusion power plant safety and regulation [4], with a longer term view of producing safety reports on developing principles for safety and regulation.

As discussed earlier, the hazard potential of a fusion plant is less than that of a fission plant, but there are still numerous hazards that need careful management in order to safely build, operate and ultimately decommission a fusion power plant. These activities must therefore be effectively regulated to ensure that the safety of workers and the public is not compromised and the environment protected at all times. The current set of fission safety standards and regulatory approaches already allow for a graded approach. However, the application of current fission codes, safety standards and regulatory approaches to fusion is not appropriate [4]. Fusion technologies are still evolving, and certain relatively new technologies, e.g. containment structures, will need to be developed according to standards and codes agreed by designers, regulators and operators of fusion plants. These technologies, in particular the first-of-a-kind cases, will inherently incorporate uncertainties and therefore require robust safety margins. At the same time, many safety requirements exist for similar fission applications, e.g. confinement or monitoring of radioactive materials. However, simply adopting these fission-based codes and standards presents a risk of introducing conservatism (safety margins) aimed at certain characteristics of fission plants which are not applicable or appropriate to fusion applications. These codes and standards will therefore need to be modified and adapted to consider the fusion specificities, and in some cases, new codes and standards will need to be developed. This should be conducted with regard to fusion technologies, materials specific to fusion and safety methodologies, while still based on a proportionate approach.

## Data Availability

This article has no additional data.
